# Sample selection bias due to omitting short trees for tree height estimation in forest inventories: A case study on *Pinus koraiensis* plantations in South Korea

**DOI:** 10.1371/journal.pone.0321160

**Published:** 2025-05-09

**Authors:** Joonghoon Shin, Yoonseong Chang, Kiwoong Lee, Dayoung Kim, Hee Han

**Affiliations:** 1 Department of Agriculture, Forestry and Bioresources, Seoul National University, Seoul, Republic of Korea; 2 Forest Management Division, National Institute of Forest Science, Seoul, Republic of Korea; 3 Forest Ecology Division, National Institute of Forest Science, Seoul, Republic of Korea; 4 Research Institute of Agriculture and Life Sciences, Seoul National University, Seoul, Republic of Korea; Chinese Academy of Sciences, CHINA

## Abstract

This study investigates the impact of omitting short tree data on tree height estimation in conventional forest inventories, focusing on *Pinus koraiensis* plantations in South Korea. Twenty height-diameter models were tested on both datasets: the complete data and the short tree-free data. The models were divided into Group 1 (with two model parameters) and Group 2 (with three model parameters) to examine whether the omission of short tree data affects model performance based on the number of parameters. Results demonstrated that excluding short tree data led to significant overestimation of tree height in small diameter ranges, with Group 2 models showing greater sensitivity to the omission. This omission also caused substantial variations in model rankings between the Full and short tree-free datasets, leading to specification errors and suboptimal model selection. Despite the small sample size difference, half of the Group 2 models produced non-significant parameter estimates when fitted to the short tree-free data, underscoring the influence of sample distribution on statistical outcomes. While most models maintained consistent height-diameter relationships during extrapolation, some generated unrealistic results, including negative or excessively large tree height estimates and inverse relationships in small diameter ranges. These findings emphasize the necessity of including short trees in forest inventory samples to mitigate biases in tree height estimation, which is critical for accurate biomass and carbon stock assessments.

## Introduction

Forest inventories commonly measure diameter at breast height (DBH) and tree height (HT), with DBH being straightforward, quick, and cost-effective, while HT measurement is labor-intensive, time-consuming, and costly. DBH is typically measured for all sampled trees, whereas HT is collected only for a subset of trees, referred to as sub-sampled trees. The HT and DBH data from sub-sampled trees are used to develop height-diameter (H-D) models, enabling HT estimation for the remaining trees [1–3]. This approach has been adopted in the National Forest Inventories (NFIs) of South Korea [4] and Germany [5].

South Korea’s NFI guidelines [6] designate the sub-sampled trees as “standard trees” to collect detailed measurements, including HT and height to crown base. These guidelines recommend sampling trees that provide a relatively even DBH distribution, with priority given to dominant or co-dominant trees forming the upper forest canopy. The assumption is that larger trees offer greater representativeness for forest-level metrics, similar to angle count sampling [7], which allocates more effort to measuring larger trees due to their presumed influence on volume estimates.

Accurate HT estimation is critical for deriving variables such as tree or stand volume, biomass, and carbon stocks [8–10]. Representative sampling across the entire range of DBH and HT is required to ensure reliable H-D relationships. However, the current focus on dominant canopy trees may exclude smaller trees, potentially biasing the results. This bias could distort information on HT growth patterns and limit the utility of H-D relationships derived from such samples.

Younger trees, particularly in dense stands, allocate resources toward HT growth during early development, often at the expense of diameter growth. Rapid HT growth in smaller trees may be critical for understanding stand dynamics, as lagging trees risk suppression or mortality [11]. Studies have confirmed this trend across species, including *Quercus glauca* [12], *Betula ermanii* [13], and Masson pine [14]. Similarly, Mehtätalo et al. [2] demonstrated that H-D relationships reflect these growth patterns, with small trees showing pronounced variability in HT-to-DBH ratios. Excluding such trees from samples risks omitting critical data where HT changes most rapidly.

Sampling bias introduced by focusing on upper canopy trees creates gaps in the representation of HT data for smaller DBH ranges. This issue, analogous to limited dependent variables in econometrics [15], has been shown to affect regression analyses by producing model specification errors [16,17]. Forest science studies echo these findings, demonstrating that sampling bias skews estimates of productivity, mortality, and growth trends [18,19]. Excluding small trees can significantly distort H-D relationships, as highlighted by Curtis and Marshall [20], who emphasized the importance of sampling trees across all size classes to define H-D curves accurately.

Omitting small trees undermines the accuracy of HT estimation for smaller and suppressed trees. Weiskittel et al. [21] and Yuancai and Parresol [22] stressed that training data must include smaller trees to ensure reliable HT estimates for biomass and carbon stock calculations. Few studies, however, have systematically examined the impacts of omitting small trees on H-D modeling. This study addresses this gap by evaluating the effects of excluding short-tree data on HT estimation in *Pinus koraiensis* plantations in Gangwon Province, South Korea. The analysis underscores the importance of inclusive sampling strategies to improve forest inventory data and enhance ecological assessments.

## Materials and methods

### Study site and tree data

This study analyzed data from 570 sample trees measured for HT and DBH within four 40m x 40m sample plots in *Pinus koraiensis* plantations in Chuncheon, Gangwon Province, South Korea (37°52´N, 127°52´E). The plantations, established in the 1960s, were surveyed in 2005 before any thinning operations. Access to the study site was granted by the Chuncheon National Forest Station. The area’s average annual temperature from 1966 to 2005 was 10.9°C, with average annual precipitation of 1,305 mm [23].

**Fig 1:**
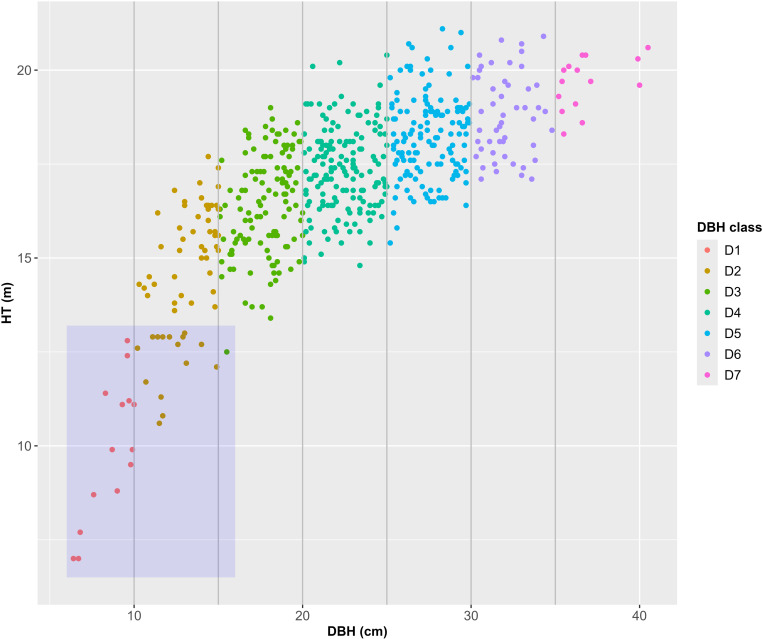
Scatterplot of the H-D relationship from the data in the study site. Observations in the blue box represent short trees (ST), with HT ≤ 13.0m. Gray vertical lines indicate DBH class boundaries.

HT measurements were conducted using a Vertex instrument (Haglöf), with two readings taken from different locations for each tree. The average of these measurements was recorded. If discrepancies between the two readings were too large, a third measurement was taken from another location, and the average of all three was used to minimize errors. Fig 1 shows the height-diameter scatterplot derived from the data. Trees were grouped into seven DBH classes following the conventions of general forest inventory. The smallest class, D1, included trees with DBH ≤ 10 cm, while the largest class, D7, encompassed trees with DBH > 35 cm. Intermediate classes were grouped in 5 cm DBH intervals.

ST, defined as those with HT ≤ 13.0 m, were identified within the blue box in Fig 1, representing approximately 5% of the total sample. These trees were assumed to be excluded from HT measurements in standard forest surveys. The dataset excluding ST was labeled “short tree-free” (STF), while the dataset including all trees was referred to as “Full.” For both datasets, the correlation coefficient between DBH and HT was significantly different from zero (t-test, α = 0.05). Descriptive statistics for the Full, STF, and ST datasets are summarized in Table 1.

**Table 1:** Descriptive statistics of the Full dataset (all trees), the STF dataset (short tree-free), and the ST dataset (short trees only).

Data	Number of trees	DBH (cm)				HT (m)			
		Avg^a^	Min^b^	Max^c^	Sd^d^	Avg^a^	Min^b^	Max^c^	Sd^d^
Full	570	22.6	6.4	40.5	6.4	17.1	7.0	21.1	2.1
STF	540	23.3	10.3	40.5	5.8	17.4	13.4	21.1	1.5
ST	30	10.6	6.4	15.5	2.3	11.2	7.0	13.0	1.8

^a^Avg=Average;

^b^Min=Minimum;

^c^Max=Maximum;

^d^Sd=Standard deviation.

Table 2 presents the variation in HT and DBH, along with the ratio of HT variation to DBH variation, categorized by DBH class. The standard deviation of HT and the ratio progressively decrease with increasing DBH class, suggesting that the sampled trees prioritized HT growth during the early stages of development.

**Table 2:** Standard deviation of HT and DBH, and the ratio of HT standard deviation to DBH standard deviation by DBH class.

	D1	D2	D3	D4	D5	D6	D7
SD_HT_^a^	1.88	1.73	1.33	1.11	1.11	1.09	0.70
SD_DBH_^b^	1.29	1.47	1.35	1.45	1.41	1.30	1.80
SD_HT_/ SD_DBH_	1.46	1.17	0.99	0.77	0.79	0.83	0.40

^a^SD_HT_=Standard deviation of HT;

^b^SD_DBH_=Standard deviation of DBH.

### Models for H-D relationship

The analysis evaluated twenty H-D models (Table 3) that use DBH to estimate HT. To account for the potential variation in the impact of excluding ST across different models, a wide range of models from previous studies was included. Models 1–9, consisting of two parameters, are categorized as “Group 1.” Models 10–20, with three parameters, are categorized as “Group 2.” Among these, only Model 1 is linear, while the remaining models are nonlinear.

**Table 3:** H-D models analyzed in this study.

Number	Model name	Equation form^a^	References
1	Linear-logarithmic	(HT−BH)=a+b ln(D)	[24]
2	Power	$(HT-BH)=aD^{b}$	[2,25]
3	Modified Curtis	$(HT-BH)=\frac{aD}{(1+D{)}^{b}}$	[2]
4	Curtis	$(HT-BH)=a{\left(\frac{D}{1+D}\right)}^{b}$	[24,25]
5	Schumacher	$(HT-BH)=a\;exp\left(-bD^{-1}\right)$	[2,25,26]
6	Meyer	(HT−BH)=a[1−exp(−bD)]	[2,25,27]
7	Näslund	$(HT-BH)={\left(\frac{D}{a+bD}\right)}^{2}$	[2,25,28]
8	Michaelis-Menten	$(HT-BH)=\frac{aD}{b+D}$	[2,25,29]
9	Wykoff	$(HT-BH)=exp\left(a-\frac{b}{1+D}\right)$	[2,25,30]
10	Ratkowsky	$(HT-BH)=a\;exp\left(\frac{-b}{c+D}\right)$	[2,25,31]
11	Schnute	$HT={\left\{{BH}^{b}+\left(c^{b}-{BH}^{b}\right)\times \frac{1-exp\left[-a\left(D-D_{1}\right)\right]}{1-exp\left[-a\left(D_{2}-D_{1}\right)\right]}\right\}}^{\frac{1}{b}}$	[25,32]
12	Chapman-Richards	$(HT-BH)=a{\left[1-exp(-bD)\right]}^{c}$	[2,25,33]
13	Weibull	$(HT-BH)=a\left[1-exp\left(-bD^{c}\right)\right]$	[2,25,34]
14	Gompertz	(HT−BH)=a exp[−b exp(−cD)]	[2,25,35]
15	Prodan	$(HT-BH)=\frac{D^{2}}{a+bD+cD^{2}}$	[2,25]
16	Sibbesen	$(HT-BH)=aD^{bD^{-c}}$	[2,25,36]
17	Logistic	$(HT-BH)=\frac{a}{\left[1+b\;exp(-cD)\right]}$	[2,25]
18	Hossfeld IV	$(HT-BH)=\frac{a}{1+b^{-1}D^{-c}}$	[2,25]
19	Lundqvist-Korf	$(HT-BH)=a\;exp\left(-bD^{-c}\right)$	[2]
20	Larsen and Hann	$(HT-BH)=exp\left(a+bD^{c}\right)$	[25,37]

^a^HT = total height (m); D = diameter at breast height (cm); BH = breast height (1.2 m above the ground); a, b, c = parameters to be estimated, D1 = 0.0 cm, D2 = 40.5 cm.

### Performance measures for the H-D models

The changes in HT estimation performance from Full to STF datasets were analyzed to assess the impact of excluding ST from the sample on HT estimation. Performance measures included bias, root mean squared error (RMSE), and the coefficient of determination (R²). The formulas for these metrics are presented below:


$$\begin{array}{c} \begin{array}{c} Bias=\frac{1}{n}\sum_{i=1}^{n}\left(y_{i}-{\hat{y}}_{i}\right)\; \end{array} \end{array}$$ (1)

$$\begin{array}{c} \begin{array}{c} RMSE=\sqrt{\frac{1}{n}\sum_{i=1}^{n}{\left(y_{i}-{\hat{y}}_{i}\right)}^{2}}\; \end{array} \end{array}$$ (2)

$$\begin{array}{c} \begin{array}{c} R^{2}=1-\frac{\sum_{i=1}^{n}{\left(y_{i}-{\hat{y}}_{i}\right)}^{2}}{\sum_{i=1}^{n}{\left(y_{i}-{\overset{―}{y}}_{i}\right)}^{2}}\; \end{array} \end{array}$$ (3)

where n is the number of observations in a given data type, $y_{i}$ is a measured HT, ${\hat{y}}_{i}$ is an estimated HT, and $\overset{―}{y}$ is the average HT in a given data type. In addition to these measures, residual plots were examined to assess model fit, error heteroscedasticity, and the presence of outliers.

### Hypothesis testing and evaluation data types

The HT estimates obtained from models fitted to the Full data were compared to those from models fitted to the STF data to determine whether the omission of ST significantly affected HT estimates. Two statistical tests were used for this comparison: Welch’s t-test and Yuen’s trimmed mean test. In this study, two evaluation types were defined. The first, referred to as Full evaluation, involved assessing models fitted to the Full data using the Full dataset, serving as the criterion for model selection. The second, called STF evaluation, assessed models fitted to the STF data using the STF dataset to examine how the omission of ST influenced the selection of the optimal model.

### Extrapolation properties of the H-D models

Extrapolation involves predicting values outside the range of the sample data used for model fitting. For reliable extrapolation, it is necessary to ensure that the relationship represented by the model remains valid beyond the sample range [38]. Previous studies have emphasized the importance of certain characteristics for HT extrapolation, including monotonic increases, the presence of an inflection point, and an upper asymptote [22]. To assess these properties, the H-D curves for each model were plotted across an expanded DBH range of 0.1 to 80 cm. These curves were examined for their ability to maintain a reasonable shape outside the sample range, exhibit monotonic increases, include an inflection point, and demonstrate an upper asymptote. The equations used to calculate the inflection point and asymptote for each model are presented in S1 Table.

### Analysis tools

All analyses were conducted using the statistical software R (version 4.2.1) [39] in the integrated development environment R Studio (version 2022.7.0.548) [40]. Linear regressions were performed using the *lm* function, and nonlinear regressions were conducted with the *nls* function without applying weights. Welch’s t-test was implemented using the *t.test* function, and Yuen’s trimmed mean test was performed with the *yuen.t.test* function from the *PairedData* package [41]. Data manipulation and graphical outputs were facilitated by the *tidyverse* package [42]. The inflection point for Model 16 was approximated using the *numDeriv* package [43], and the *Ryacas* package [44] was used to solve cubic equations for calculating the inflection point of Model 15.

## Results

### Changes in estimates of model parameters

The parameter estimates for the models, categorized by data type, are presented in Table 4. Group 2 models exhibited relatively larger changes in parameter values compared to Group 1 models, depending on the dataset used. For the Full data, most parameters were statistically significant at α = 0.05. In contrast, the STF data showed a higher frequency of non-significant parameters. Specifically, in models 13, 18, 19, and 20, all three parameters were non-significant, while in model 12, two out of three parameters were non-significant.

**Table 4:** Parameter estimates for the H-D equations by model and data type.

Model	Full data			STF data		
a	b	c	a	b	c
1	-0.3374^*^	5.2801		3.9406	3.9428	
2	5.7235	0.3307		7.5242	0.2460	
3	6.6203	0.7058		8.7002	0.7893	
4	21.9009	6.8817		20.6160	5.3883	
5	21.6919	6.5042		20.4855	5.1214	
6	18.4478	0.0956		17.9128	0.1096	
7	0.8346	0.2113		0.6481	0.2189	
8	23.2977	9.9043		21.3368	6.9818	
9	3.0963	7.2768		3.0326	5.6675	
10	20.1647	4.0566	-3.3383	25.3773	18.2462	18.1267
11	0.1372	0.4334	18.8560	0.0026^*^	4.2401	19.8652
12	17.7529	0.1372	1.6819	30.2813^*^	0.0041^*^	0.2577
13	17.7666	0.0594	1.2096	49.3218^*^	0.1555^*^	0.3018^*^
14	17.5915	2.3726	0.1582	20.4985	0.6886	0.0476
15	3.7043	0.0144^*^	0.0532	-1.9320	0.5197	0.0424
16	18.8139	-27.5989	2.0621	30.6198	-1.8672	0.7103
17	17.4765	3.8237	0.1837	20.0772	0.8930	0.0584
18	18.5403	0.0119	2.0735	75.0334^*^	0.1038^*^	0.3133^*^
19	19.0533	22.4175	1.6015	468.2622^*^	4.2226^*^	0.0730^*^
20	2.9472	-22.4176	-1.6015	6.1494^*^	-4.2229^*^	-0.0730^*^

* Not significant at α=0.05.

The asymptotic parameters of models 11, 12, 13, 18, 19, and 20, estimated from the STF data, were noticeably higher compared to those estimated from the Full data. All these parameters from the STF data were non-significant. Changes in the sign of parameter estimates were observed for *a* in models 1 and 15 and for *c* in model 10.

### Changes in HT estimates

**Fig 2:**
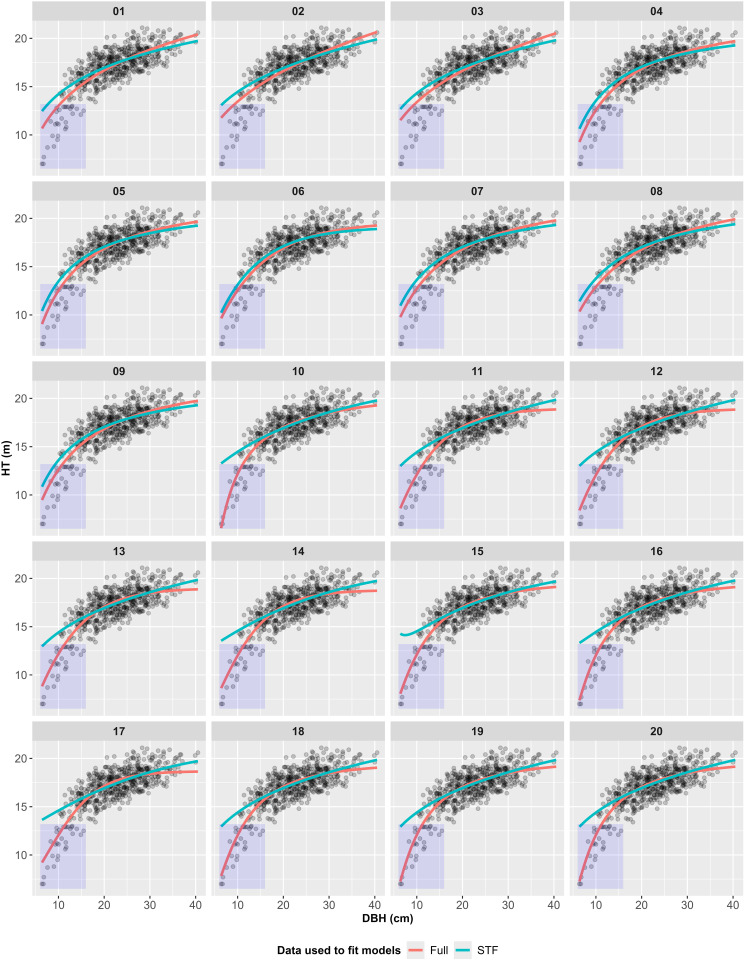
H-D curves by model and data type (the DBH ranges 6.4 to 40.5 cm).

Fig 2 illustrates the H-D curves for each model, categorized by data type, alongside the data points used for model fitting. Changes in HT estimates resulting from the omission of ST were generally observed at both ends of the curves. The extent and pattern of these changes differed between Group 1 and Group 2 models.

The models in Group 1 (models 1–9) exhibited smaller changes in HT estimates due to the omission of ST compared to those in Group 2 (models 10–20). In Group 1, HT estimates slightly increased in the small DBH range and slightly decreased in the large DBH range. Welch’s t-test (α = 0.05) identified significant changes in HT estimates between the Full and STF datasets only for models 2 and 3. In contrast, Yuen’s trimmed mean test found no significant differences between the datasets. Although the overall shape of the H-D curves remained largely unchanged, the curves appeared slightly rotated clockwise. Among Group 1 models, model 6 was the least affected by the omission of ST.

In Group 2, the omission of ST led to more pronounced shifts in HT estimates. When switching from the Full data to the STF data, HT estimates significantly increased in the small DBH range, resulting in severe overestimation, and slightly increased in the large DBH range. Welch’s t-test (α = 0.05) indicated significant changes in HT estimates for all Group 2 models, whereas Yuen’s trimmed mean test identified significant differences only for models 14 and 17. The H-D curves generated by Group 2 models underwent more noticeable changes in shape, with curves derived from the STF data appearing smoother and closer to a straight line compared to those from the Full data.

**Fig 3:**
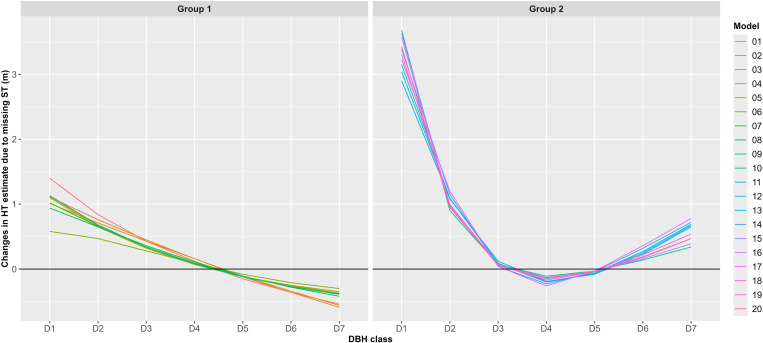
Changes in HT estimate due to omitting ST by DBH class, model, and model group.

The impact of omitting ST on HT estimates becomes clearer when analyzed by DBH class (Fig 3). In Group 1, the changes in HT estimates between the Full and STF datasets increased by approximately 1 m in the D1 class. As DBH class increased, these changes gradually diminished, becoming negative from the D5 class onward. In Group 2, the only DBH classes where HT estimates decreased were D4 and D5, located in the middle of the DBH classes. In contrast, HT estimates increased for all other classes. Notably, in the D1 class, HT estimates in Group 2 increased by about 3 m, which was three times the change observed in Group 1 for the same class. Similarly, in the D2 class, HT estimates in Group 2 increased by about 1 m.

### Performance measures and evaluation ranks

The performance of the H-D models and their rankings across data types are summarized in Table 5. For the Full data, Group 2 models generally outperformed those in Group 1. Among individual models, model 10 demonstrated the best performance based on R² and RMSE, followed by models 20 and 19. The poorest performances were observed in models 2, 3, and 1, in that order.

**Table 5:** Performance and performance ranking of H-D equations by model and data type.

Model	R^2^		RMSE (m)^b^		Bias (m)	
	Full	STF	Full	STF	Full^c^	STF^c^
1	0.6304 (18)^a^	0.4615 (11)	1.2608	1.1144	0.0000 (1)	-0.0000 (1)
2	0.6044 (20)	0.4618 (8)	1.3044	1.1142	-0.0037 (16)	-0.0000 (11)
3	0.6129 (19)	0.4619 (1)	1.2903	1.1141	-0.0054 (18)	0.0000 (12)
4	0.6597 (11)	0.4528 (18)	1.2098	1.1234	-0.0028 (14)	0.0004 (16)
5	0.6621 (9)	0.4518 (19)	1.2055	1.1245	-0.0027 (13)	0.0004 (17)
6	0.6567 (14)	0.4369 (20)	1.2153	1.1396	-0.0061 (20)	0.0034 (20)
7	0.6536 (15)	0.4544 (16)	1.2208	1.1218	-0.0043 (17)	0.0005 (18)
8	0.6444 (17)	0.4565 (15)	1.2368	1.1196	-0.0059 (19)	0.0006 (19)
9	0.6571 (13)	0.4537 (17)	1.2144	1.1225	-0.0029 (15)	0.0004 (15)
10	0.6745 (1)	0.4615 (10)	1.1832	1.1144	0.0000 (2)	-0.0000 (5)
11	0.6623 (8)	0.4618 (6)	1.2052	1.1141	-0.0021 (10)	-0.0000 (8)
12	0.6632 (7)	0.4618 (7)	1.2036	1.1141	-0.0013 (7)	-0.0000 (9)
13	0.6609 (10)	0.4618 (5)	1.2078	1.1141	-0.0026 (12)	-0.0000 (7)
14	0.6595 (12)	0.4611 (12)	1.2103	1.1149	-0.0013 (6)	-0.0000 (10)
15	0.6696 (6)	0.4611 (13)	1.1921	1.1149	-0.0020 (9)	0.0001 (14)
16	0.6719 (4)	0.4616 (9)	1.1880	1.1144	-0.0006 (5)	0.0000 (3)
17	0.6530 (16)	0.4608 (14)	1.2217	1.1152	-0.0024 (11)	-0.0000 (13)
18	0.6697 (5)	0.4618 (4)	1.1919	1.1141	-0.0014 (8)	-0.0000 (6)
19	0.6726 (3)	0.4619 (3)	1.1867	1.1141	-0.0005 (4)	-0.0000 (2)
20	0.6726 (2)	0.4619 (2)	1.1867	1.1141	-0.0005 (3)	-0.0000 (4)

^a^The numbers in parentheses indicate the performance ranking of an H-D equation based on the corresponding model, performance measure, and dataset.

^b^The models’ ranks for RMSE are all the same as the ranks for R^2^.

^c^All the models from the Full and STF data type were unbiased based on both Welch’s t-test and Yuen’s trimmed mean test at α=0.05.

In terms of bias, model 1 from Group 1 exhibited the best performance, while the remaining top-ranked models were from Group 2. However, the differences in bias among models were minimal. Bias tests confirmed that all models were unbiased. Despite being part of Group 2, model 17 consistently ranked in the middle or lower range across data types and performance measures, showing relatively weaker performance compared to its group counterparts.

When using the STF data, R² and RMSE values were lower than those obtained with the Full data (Table 5). Despite these changes, all models remained unbiased in terms of Bias. Performance differences among models narrowed significantly when using the STF data. Model rankings based on R² and RMSE shifted noticeably, with model 3 achieving the highest performance, followed by models 20 and 19.

The rankings based on R² and RMSE varied substantially depending on the dataset. Model 3 showed the most dramatic improvement, rising from 19th place with the Full data to 1st place with the STF data. In contrast, model 10, which ranked 1st with the Full data, dropped to 10th with the STF data. Models 19 and 20 consistently maintained high rankings across both datasets, reflecting their robustness to the omission of ST.

**Fig 4:**
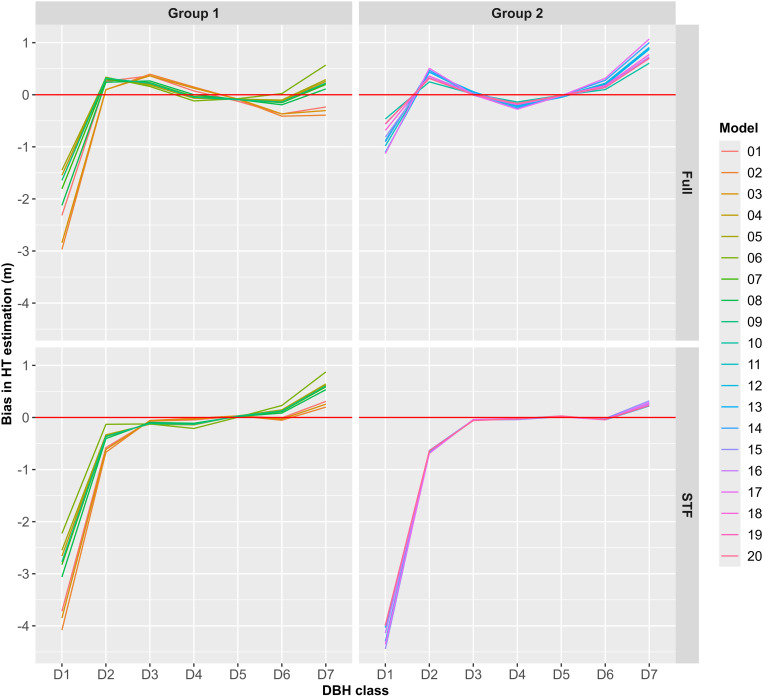
Bias in HT estimation by DBH class, model, model group, and data type.

Fig 4 highlights the Bias in HT estimation by DBH class, model, model group, and data type. The most pronounced impact of omitting ST was observed in the D1 class, where overestimation in the STF data significantly increased compared to the Full data. In Group 1, overestimation in the D1 class increased by approximately 1 m, while in Group 2, it increased by about 3 m. In other DBH classes, the changes in HT estimation were relatively small. However, in the D2 class, underestimation observed in the Full data shifted to overestimation in the STF data. Another notable effect of omitting ST was the reduction in differences in HT estimation among models within Group 2, leading to more uniform predictions across these models.

Residual plots for models fitted with the Full data (S1 Fig) showed no evidence of poor fit or systematic patterns indicative of model specification errors. Models 1–3 exhibited relatively large overestimations for ST, but errors for most models satisfied the assumption of homoscedasticity. In contrast, residual plots for models fitted with the STF data (S2 Fig) revealed overestimation for ST across all models, violating the assumption of homoscedasticity. However, this overestimation was less pronounced in Group 1 models (models 4–9). Apart from the overestimation of ST, no additional patterns suggested model specification errors in either dataset.

### Extrapolation properties of H-D models

**Fig 5:**
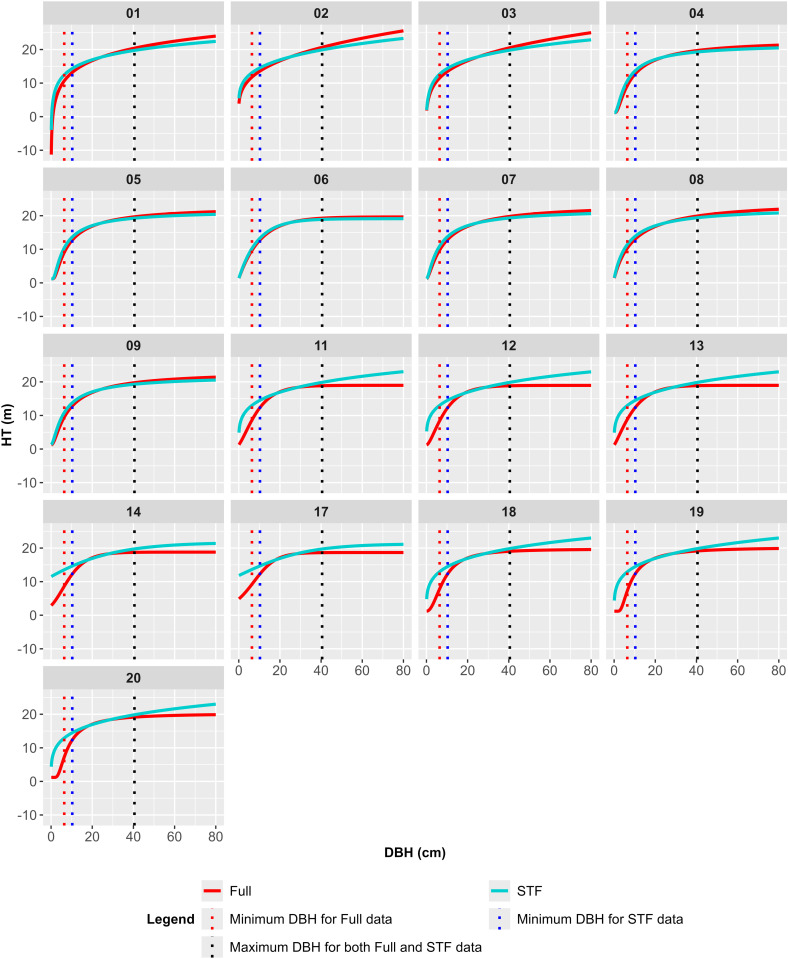
Extrapolated H-D curves by model (excluding models 10, 15, and 16) and data type (DBH ranges: 0.1 to 80.0 cm).

The H-D curves generated by the estimated models for the expanded DBH range of 0.1 to 80 cm are shown for each data type in Fig 5. Red dashed lines represent the minimum DBH value for the Full data, while blue dashed lines indicate the minimum DBH value for the STF data. Black dashed lines denote the maximum DBH value common to both datasets. Curve segments falling outside the minimum or maximum DBH values of the respective dataset are considered extrapolation.

Models 10, 15, and 16 produced exceptionally large HT estimates in the small DBH range, complicating visual comparisons on the same plot. As a result, these models are presented separately in dedicated figures to allow for clearer examination.

The omission of ST did not significantly affect the extrapolation properties of Group 1 models (Fig 5). However, model 1 produced negative HT estimates within a very small DBH range, indicating limitations in its extrapolation behavior. Models 1–3, which lack an asymptote (Table 6), showed a relatively large decrease in HT estimates at DBH = 80 cm when ST was omitted. Despite these changes, all models in Group 1 maintained a monotonic increase across the expanded DBH range for both the Full and STF datasets.

**Table 6:** Calculated inflection points and asymptotes of the H-D models analyzed.

Model number	Inflection point calculated^a^		Asymptote calculated	
Full	STF	Full	STF
1	–	–	–	–
2	–	–	–	–
3	–	–	–	–
4	(2.9, 4.1)	(2.2, 3.9)	23.10	21.82
5	(3.3, 4.1)	(2.6, 4.0)	22.89	21.69
6	–	–	19.65	19.11
7	(2.0, 3.7)	(1.5, 3.5)	23.60	22.07
8	–	–	24.50	22.54
9	(2.6, 4.2)	(1.8, 4.0)	23.32	21.95
10	(5.4, 3.9)	–	21.36	26.58
11	(3.5, 2.0)	–	18.97	34.08
12	(3.8, 8.4)	–	18.95	31.48
13	(2.4, 4.0)	–	18.97	50.52
14	(5.5, 7.7)	–	18.79	21.70
15	(4.8, 5.8)	(12.3, 15.1)	20.00	24.78
16	(5.2, 5.3)	(7.9, 13.8)	20.01	31.82
17	(7.3, 9.9)	–	18.68	21.28
18	(5.1, 6.0)	–	19.74	76.23
19	(5.1, 5.0)	(0.0, 1.2)	20.25	469.46
20	(5.1, 5.0)	(0.0, 1.2)	20.25	469.62

^a^The inflection point calculated is expressed as (DBH, HT).

Inflection points in Group 1 were observed only in models 4, 5, 7, and 9 (Table 6). For these models, the inflection points fitted to the Full data were located near the left tails of the H-D curves. With the omission of ST, these inflection points shifted slightly closer to the origin, reflecting minor changes in curve shape.

Group 2 models were more affected than Group 1 models by the omission of ST, particularly at the tails of the H-D curves. Except for models 10, 15, and 16, Group 2 models fitted to the Full data exhibited characteristics of monotonic increase (Fig 5), inflection points, and asymptotes (Table 6). In the STF data, all Group 2 models maintained monotonic increases (Fig 5), with inflection points observed only in models 19 and 20, located very close to the origin (Table 6). The asymptotes increased for all models, with models 13 and 18–20 producing unrealistically large asymptote values.

**Fig 6:**
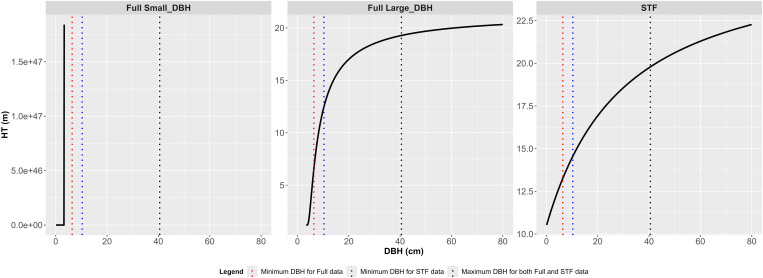
Extrapolated H-D curve of Model 10 by data type (0 < Full_Small DBH < 3.4 cm, 3.4 ≤ Full_Large DBH ≤ 80 cm, 0 < STF ≤ 80 cm).

Model 10 displayed non-monotonic increases and abnormal HT estimates in the small DBH range below 3.3 cm in the Full data (“Full Small_DBH” in Fig 6). For DBH values above 3.4 cm, model 10 exhibited an S-shaped curve (“Full Large_DBH” in Fig 6), demonstrating characteristics of monotonic increase, an inflection point, and an asymptote (Table 6). When fitted to the STF data, the curve for model 10 became concave, lacking an inflection point and an asymptote (“STF” in Fig 6). Model 10 also generated an abnormal HT estimate of over 10 m at DBH = 0.1 cm, leading to overestimation in the small DBH range regardless of the dataset used.

**Fig 7:**
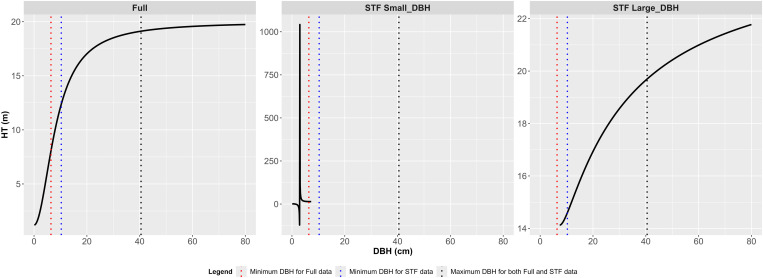
Extrapolated H-D curve of Model 15 by data type (0 cm < Full ≤ 80 cm, 0 cm < STF_Small DBH < 7.4 cm, 7.4 cm ≤ STF_Large DBH ≤ 80 cm).

Model 15 exhibited an S-shaped curve with characteristics of monotonic increase, an inflection point, and an asymptote when fitted to the Full data (“Full” in Fig 7). However, when fitted to the STF data, the model produced abnormal results. In the DBH range of 3.1 to 7.4 cm, it generated excessively large HT estimates without a monotonic increase. In the DBH range below 3 cm, the model yielded negative HT estimates (“STF Small_DBH” in Fig 7). For DBH values above 7.4 cm, the curve displayed a monotonic increase and an inflection point, but the asymptote was outside this DBH range (“STF Large_DBH” in Fig 7, Table 6).

**Fig 8:**
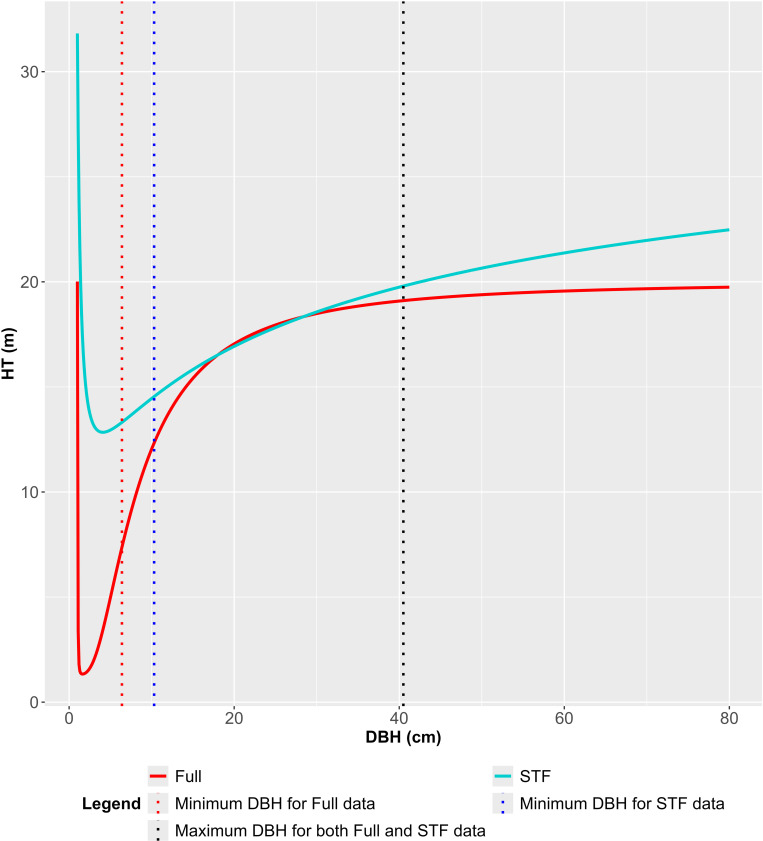
Extrapolated H-D curve of Model 16 by data type (DBH range: 1.0 cm to 80.0 cm).

Model 16’s H-D curves are presented in Fig 8. Although the DBH range depicted in Fig 8 excludes the smallest values, model 16 produced abnormally large HT estimates in the DBH range of 0.1 to 1.0 cm, regardless of the dataset. These anomalous estimates prevented the observation of monotonic increases within specific DBH ranges (Full data: 0.1 to 1.6 cm; STF data: 0.1 to 4.1 cm). For the STF dataset, the curve did not show its estimated asymptote of 31.82 cm within the expanded DBH range depicted in Fig 5, indicating a lack of practical asymptotic behavior.

Remarkable changes in extrapolation for larger trees due to the omission of ST were observed in Group 2 models (Table 7). As DBH approached 80.0 cm, beyond the maximum DBH value of the sample data, the differences between HT estimates from the two datasets increased significantly for Group 2 models (Figs 5–8). Table 7 summarizes the differences in HT estimates at DBH values of 40.5 and 80.0 cm by model. At DBH = 40.5 cm, Group 1 models showed absolute differences ranging from 0.4 to 0.8 m, while Group 2 models exhibited differences ranging from 0.5 to 1.1 m. At DBH = 80.0 cm, the differences were more pronounced for Group 2 models, ranging from 1.9 to 4.1 m, compared to Group 1’s range of 0.5 to 2.3 m.

**Table 7:** Changes in HT extrapolations due to omitting ST at DBH = 40.5 and 80.0 cm by model.

Model	1	2	3	4	5	6	7	8	9		
Change^a^ at DBH = 40.5 cm	-0.7	-0.8	-0.7	-0.4	-0.4	-0.4	-0.5	-0.5	-0.5		
Change at DBH = 80.0 cm	-1.6	-2.3	-2.1	-0.8	-0.8	-0.5	-0.9	-1.1	-0.9		
Model	10	11	12	13	14	15	16	17	18	19	20
Change at DBH = 40.5 cm	0.5	1.0	1.0	1.0	1.0	0.6	0.7	1.1	0.8	0.7	0.7
Change at DBH = 80.0 cm	1.9	4.1	4.0	4.0	2.6	2.0	2.7	2.4	3.4	3.1	3.1

^a^Change = HT estimated from the STF data - HT estimated from the Full data.

## Discussion

### Impact of omitting ST on model performance and parameter estimates

This study examined how the omission of ST impacts HT estimation in *Pinus koraiensis* plantations. Omitting ST from the dataset generally led to overestimation of HT for ST. The degree of this overestimation varied by model group, with Group 2 models being more affected than Group 1 models. The omission influenced parameter estimates, HT predictions, and model performance, often resulting in model specification errors, where suboptimal models were selected as the final models.

Non-significant parameter estimates due to ST omission were observed exclusively in some Group 2 models (Table 4). Huang et al. [25] noted that statistical significance is often better in two-parameter models than in three-parameter models, particularly in small datasets. However, the dataset sizes in this study—570 for Full data and 540 for STF data—are considerably larger than the datasets cited in Huang et al. [25], where sample sizes ranged from 102 to 135. The relatively small difference of 30 observations between the Full and STF datasets suggests that both sample size and distribution play critical roles in determining the significance of parameter estimates. These findings align with [45], who reported that limited DBH and HT ranges or the absence of asymptotic trends can lead to unrealistically large asymptotic parameter estimates.

The severe overestimation of HT in the small DBH range due to ST omission, as illustrated in Fig 4 and S2 Table, corroborates findings by Weiskittel et al. [21], who highlighted significant errors in HT estimation for ST when ST is excluded from model training. Additionally, this study identified notable differences in the extent of this impact between model groups, with Group 2 showing much larger overestimations. The greater sensitivity of Group 2 to ST omission likely stems from the increased flexibility afforded by its additional parameters, which, while beneficial under ideal conditions, may exacerbate errors under biased sampling conditions. This flexibility, often considered a strength of complex models [25], paradoxically contributed to poorer performance in estimating HT for ST when sampling bias was introduced.

The changes in model performance observed here cannot be attributed solely to model complexity. Sampling bias, overfitting, and increased sensitivity associated with model complexity may act independently or interactively. While more complex models are typically associated with reduced bias and increased variance, Group 2 models did not consistently exhibit lower bias than Group 1 models within the same dataset (Table 5). Similarly, Group 2 models did not always show higher variance in estimates compared to Group 1 models (S3 Table). This complexity underscores the importance of evaluating models not just based on the number of parameters but also their “flexibility to data,” a concept deserving further exploration in future research.

### Model performance rankings and hypothesis testing

The omission of ST led to significant changes in model performance rankings, particularly for R² and RMSE (Table 5). For example, model 3 shifted from 19th place with Full data to 1st place with STF data, illustrating the influence of sample selection bias on model specification. Conversely, models 18, 19, and 20 maintained high rankings across both datasets, indicating greater robustness to ST omission.

Hypothesis tests further highlighted the impact of ST omission. Welch’s t-test indicated significant differences in HT estimates for models 2, 3, and all Group 2 models. However, the more robust Yuen’s test identified differences only in models 14 and 17. This variability underscores the importance of the testing method used. Consistent with [46], this study observed significant changes in H-D relationships at the extremes of the data range (Figs 2 and 3). Hypothesis tests conducted by DBH class revealed significant differences in most cases, except for classes D3 and D5 in Group 2, where both tests produced nearly identical results (S4 Table).

### Extrapolation characteristics and robustness of models

The superior performance of Group 2 models with the Full dataset (Table 6) aligns with findings from [25], which reported that three-parameter models generally outperform two-parameter models. Similarly, Mehtätalo et al. [2] observed that three-parameter models performed comparably to or slightly worse than two-parameter models in terms of RMSE.

For the STF dataset, RMSE values unexpectedly decreased, becoming smaller than those for the Full dataset. This result is misleading because the variability of HT values in the STF dataset (standard deviation 1.5) was only 71.4% of that in the Full dataset (standard deviation 2.1). Reduced variability in the dependent variable artificially improved RMSE while coinciding with a decline in R², highlighting the limitation of RMSE as a sole performance metric under such conditions.

When Group 2 models were fitted to the STF data, HT estimates sharply decreased as DBH approached 0 cm, falling outside the range of training data (Fig 3). Models 10, 14, and 17 deviated from this trend, yielding HT estimates exceeding 10 m near DBH = 0 cm (Figs 3 and 4). Additionally, five models, including models 15 and 16, produced extreme HT estimates in the small DBH range (Figs 5 and 6), suggesting they were less robust to ST omission despite maintaining reasonable estimation performance for ST.

Previous studies often classify H-D models by the number of parameters, but none have explored how omitting ST impacts model behavior based on parameter count. Existing research highlights the issues arising from excluding small trees during training, such as difficulties in estimating the height of small trees or undefined shapes of the H-D curve in the small tree segment. However, there has been little discussion on how such omissions affect HT estimates for larger trees. This study demonstrates that omitting small trees can significantly impact extrapolation for larger trees in certain models.

Curtis and Marshall [20] emphasized that for developing H-D curves, about two-thirds of sampled trees should come from those larger than the stand’s average DBH, with the remaining one-third from smaller trees. However, because young or small trees prioritize height growth over diameter growth, an inadequate number of samples from the small DBH range may result in poor representation of this segment. Sampling criteria should, therefore, explicitly consider height distribution, especially for smaller DBH ranges, to ensure well-represented H-D relationships.

Differences between model groups were also evident in extrapolation behavior for large DBH ranges. At DBH = 80 cm, absolute differences in height estimates between Full and STF datasets ranged from 1.9 to 4.1 m for Group 2 models, about 2.5 times greater than those for Group 1 (Table 7). Models 13, 18, 19, and 20 had asymptotic values far exceeding the maximum height of 30 m reported for Korean pine in South Korea [47]. Although these models did not exhibit significant differences in their fit to training data, their extrapolation results suggest that asymptotic characteristics are a meaningful criterion when selecting height estimation models.

This study found that omitting ST in training data can lead to increased HT estimates for large trees in Group 2, while estimates for large trees in Group 1 tended to decrease (Table 7). Although these estimates could not be validated due to being outside the training data range, significant differences between Full and STF datasets were observed near DBH = 80 cm. Weiskittel et al. [21] and Yuancai and Parresol [22] emphasized the importance of including small trees in training datasets to ensure accurate HT estimation for ST. However, this study extends their findings by showing that omitting ST also affects HT estimation for larger trees, depending on the model used.

## Conclusions

This study examined the impact of omitting ST on HT estimation in H-D models for *Pinus koraiensis* plantations in South Korea. The omission of ST led to overestimation of HT, especially for smaller trees, and had a more pronounced impact on three-parameter models. Non-significant parameter estimates were more prevalent in these models, underscoring the importance of both sample size and distribution in maintaining parameter significance.

These findings have critical implications for forest inventory practices. Including a representative range of tree sizes is essential to avoid biases in HT estimation, ensuring more accurate biomass and carbon stock calculations. Although this study highlights the consequences of ST omission, further research is needed to examine the effects across different forest growth stages [48] and validate extrapolation for larger DBH ranges.

Accurate HT estimation is crucial for forest carbon assessments. By demonstrating how sample selection biases affect HT estimation, this study emphasizes the need for comprehensive sampling strategies. Including ST in training datasets enhances the robustness and reliability of H-D relationships, improving the precision of biomass and carbon stock estimations in forest inventories.

## Supporting information

S1 Fig Residual plots of models estimated with Full data. (TIF)

S2 Fig Residual plots of models estimated with STF data. (TIF)

S1 Table Equations used to calculate inflection points and asymptotes by model. (DOCX)

S2 Table Bias in HT estimates by DBH class, data type, and model. (DOCX)

S3 Table Variance of HT estimates by model and data type. (DOCX)

S4 Table Average changes between HTs estimated from the two datasets by DBH class and model. (DOCX)
