# Supplementary figures and images for: Sample selection bias due to omitting short trees for tree height estimation in forest inventories: A case study on Pinus koraiensis plantations in South Korea
Source: PLoS One. 2025 May 9;20(5):e0321160. doi: 10.1371/journal.pone.0321160 (PMC12063842; doi:10.1371/journal.pone.0321160)

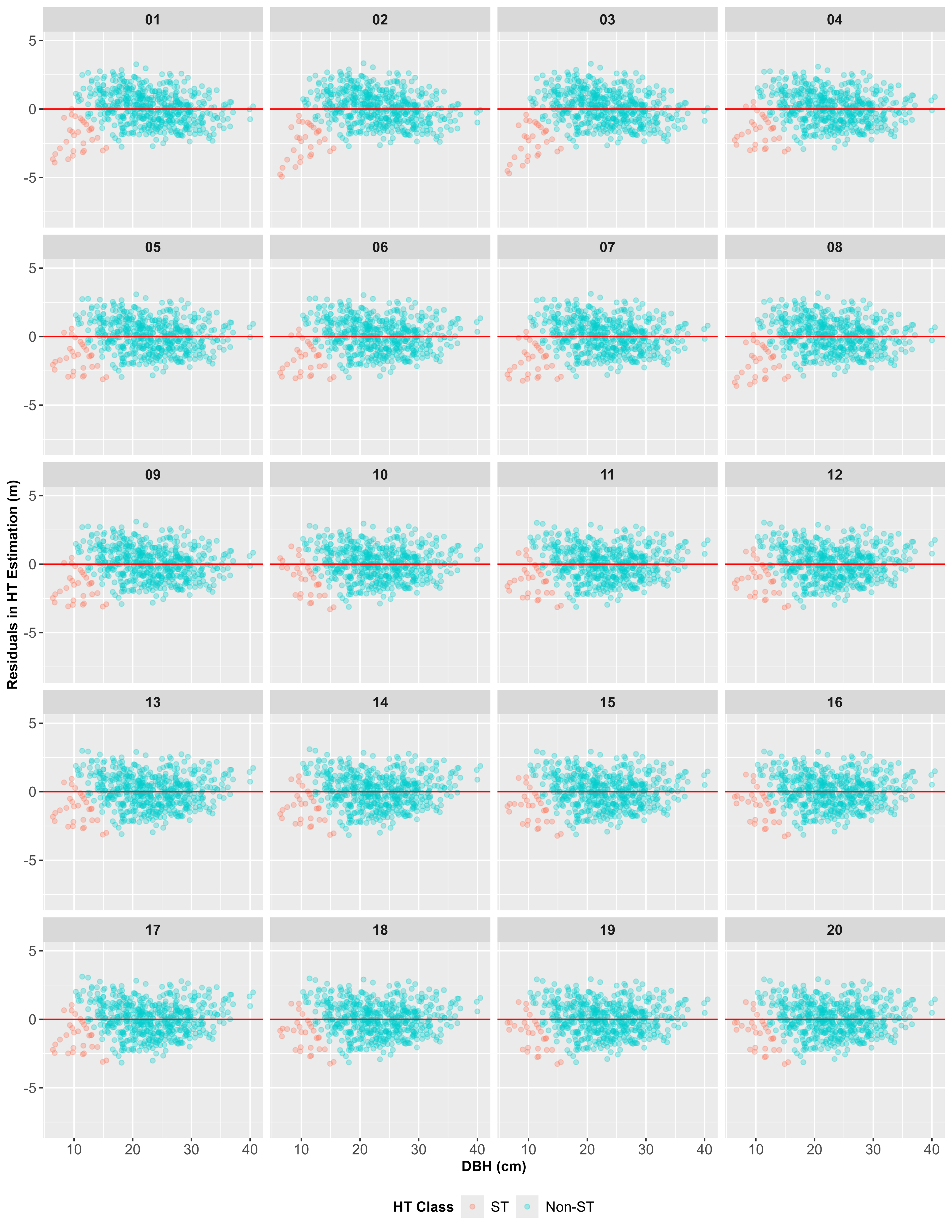

Supplement: S1 Fig — (TIF) [file pone.0321160.s001.tif]

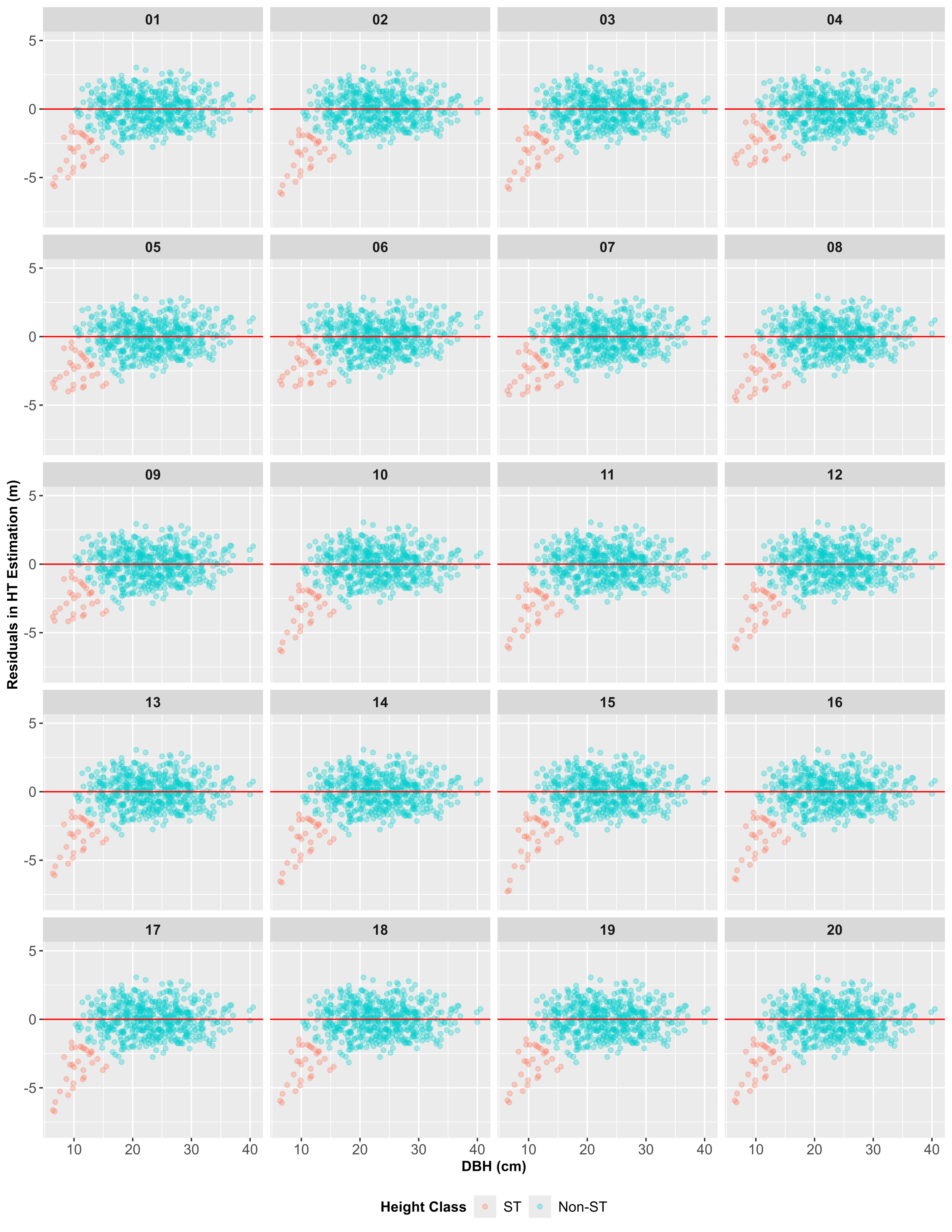

Supplement: S2 Fig — (TIF) [file pone.0321160.s002.tif]
