# Supplementary material for: Sample selection bias due to omitting short trees for tree height estimation in forest inventories: A case study on Pinus koraiensis plantations in South Korea
Source: PLoS One. 2025 May 9;20(5):e0321160. doi: 10.1371/journal.pone.0321160 (PMC12063842; doi:10.1371/journal.pone.0321160)
Supplement: S1 Table — (DOCX) [file pone.0321160.s003.docx]

S1 Table. Equations used to calculate inflection points and asymptotes by model.

| Model number | Inflection point^*^ | Asymptote^*^ |
| --- | --- | --- |
| 1 | None | $H\to\infty$ |
| 2 | None | $H\to\infty$ |
| 3 | $DBH=\frac{2}{b-1}; HT=\frac{2\left( b-1 \right)^{b-1}}{\left( b+1 \right)^{b}}+BH$ | $H\to\infty$ |
| 4 | $DBH=\frac{b-1}{2}; HT=a\left( \frac{b-1}{b+1} \right)+BH$ | $HT\to a+BH$ |
| 5 | $DBH=\frac{b}{2}; HT=\frac{a}{e^{2}}+BH$ | $HT\to a+BH$ |
| 6 | None | $HT\to a+BH$ |
| 7 | $DBH=\frac{a}{2b}; HT=\frac{1}{9b^{2}}+BH$ | $HT\to\frac{1}{b^{2}}+BH$ |
| 8 | None | $HT\to a+BH$ |
| 9 | $DBH=\frac{b}{2}-1; HT=\exp\left( a-2 \right)+BH$ | $HT\to\exp\left( a \right)+BH$ |
| 10 | $DBH=\frac{b}{2}-c; HT=\frac{a}{e^{2}}+BH$ | $HT\to a+BH$ |
| 11 | $DBH=D_{1}+D_{2}-\frac{1}{a}\ln\left[ \frac{b\left\{ \exp\left( aD_{2} \right)c^{b}-\exp\left( aD_{1} \right){BH}^{b} \right\}}{c^{b}-{BH}^{b}} \right]; HT=\left( 1-b \right)\left[ \frac{\exp\left( aD_{2} \right)c^{b}-\exp\left( aD_{1} \right){BH}^{b}}{\exp\left( aD_{2} \right)-\exp\left( aD_{1} \right)} \right]$ | $HT\to\left[ {BH}^{b}+\frac{c^{b}-{BH}^{b}}{1-\exp\left( -a\left( D_{2}-D_{1} \right) \right)} \right]^{\frac{1}{b}}$ |
| 12 | $DBH=-\frac{1}{b}\ln\left( \frac{1}{c} \right); HT=a\left[ \frac{c-1}{c} \right]^{c}+BH$ | $HT\to a+BH$ |
| 13 | $DBH=\left( \frac{c-1}{bc} \right)^{\frac{1}{c}}; HT=a\left[ 1-\exp\left( \frac{1-c}{c} \right) \right]+BH$ | $HT\to a+BH$ |
| 14 | $DBH=\frac{\ln\left( b \right)}{c}; HT=\frac{a}{e}+BH$ | $HT\to a+BH$ |
| 15 | $bc{DBH}^{3}+3ac{DBH}^{2}-a^{2}=0$ | $HT\to\frac{1}{c}+BH$ |
| 16 | Approximated by numerical derivatives | $HT\to a+BH$ |
| 17 | $DBH=\frac{1}{c}\ln\left( b \right); HT=\frac{a}{2}+BH$ | $HT\to a+BH$ |
| 18 | $DBH=\left[ \frac{c-1}{b\left( c+1 \right)} \right]^{\frac{1}{c}}; HT=\frac{a\left( c-1 \right)}{2c}+BH$ | $HT\to a+BH$ |
| 19 | $DBH=\left( \frac{bc}{c+1} \right)^{\frac{1}{c}}; HT=a \exp\left( -\frac{c+1}{c} \right)+BH$ | $HT\to a+BH$ |
| 20 | $DBH=\left( \frac{1-c}{bc} \right)^{\frac{1}{c}}; HT=\exp\left( a+\frac{1-c}{c} \right)+BH$ | $HT\to\exp\left( a \right)+BH$ |

^*^ BH: breast height, which was 1.2 m for this study, D_1_: 0.0cm, D_2_: 40.5cm
