# Supplementary material for: Sample selection bias due to omitting short trees for tree height estimation in forest inventories: A case study on Pinus koraiensis plantations in South Korea
Source: PLoS One. 2025 May 9;20(5):e0321160. doi: 10.1371/journal.pone.0321160 (PMC12063842; doi:10.1371/journal.pone.0321160)
Supplement: S2 Table — (DOCX) [file pone.0321160.s004.docx]

S2 Table. Bias in HT estimates by DBH class, data type, and model.

| Data type | Model | D1 | D2 | D3 | D4 | D5 | D6 | D7 |
| --- | --- | --- | --- | --- | --- | --- | --- | --- |
| Full | 1 | **-2.32** | 0.26 | **0.36** | 0.07 | -0.13 | **-0.37** | -0.24 |
|  | 2 | **-2.97** | 0.10 | **0.39** | 0.15 | -0.09 | **-0.41** | **-0.39** |
|  | 3 | **-2.84** | 0.10 | **0.36** | 0.13 | -0.08 | **-0.37** | -0.30 |
|  | 4 | **-1.55** | 0.32 | 0.20 | -0.06 | -0.09 | -0.12 | 0.26 |
|  | 5 | **-1.45** | 0.32 | 0.19 | -0.07 | -0.08 | -0.10 | 0.29 |
|  | 6 | **-1.64** | 0.34 | 0.16 | -0.12 | -0.08 | 0.02 | **0.57** |
|  | 7 | **-1.81** | 0.29 | **0.23** | -0.03 | -0.09 | -0.15 | 0.20 |
|  | 8 | **-2.12** | 0.24 | **0.27** | 0.00 | -0.09 | -0.19 | 0.11 |
|  | 9 | **-1.64** | 0.32 | 0.22 | -0.05 | -0.09 | -0.14 | 0.22 |
|  | 10 | -0.47 | 0.25 | 0.01 | -0.14 | -0.01 | 0.10 | **0.61** |
|  | 11 | **-0.98** | **0.43** | 0.03 | **-0.22** | -0.04 | 0.21 | **0.89** |
|  | 12 | **-0.88** | **0.43** | 0.02 | **-0.23** | -0.03 | 0.22 | **0.91** |
|  | 13 | **-1.11** | **0.43** | 0.06 | **-0.21** | -0.05 | 0.19 | **0.87** |
|  | 14 | **-0.90** | **0.47** | -0.01 | **-0.26** | -0.02 | 0.28 | **1.00** |
|  | 15 | **-0.81** | 0.36 | 0.03 | **-0.17** | -0.03 | 0.14 | **0.71** |
|  | 16 | -0.57 | 0.33 | 0.00 | **-0.18** | -0.01 | 0.16 | **0.73** |
|  | 17 | **-1.13** | **0.51** | 0.00 | **-0.28** | -0.02 | **0.32** | **1.07** |
|  | 18 | **-0.68** | 0.36 | 0.00 | **-0.19** | -0.01 | 0.18 | **0.77** |
|  | 19 | -0.56 | 0.31 | 0.00 | -0.17 | -0.01 | 0.14 | **0.70** |
|  | 20 | -0.56 | 0.31 | 0.00 | -0.17 | -0.01 | 0.14 | **0.70** |
| STF | 1 | **-3.72** | **-0.58** | -0.07 | -0.05 | 0.03 | -0.01 | 0.31 |
|  | 2 | **-4.08** | **-0.67** | -0.06 | -0.02 | 0.03 | -0.05 | 0.20 |
|  | 3 | **-3.85** | **-0.61** | -0.06 | -0.03 | 0.03 | -0.03 | 0.26 |
|  | 4 | **-2.66** | -0.35 | -0.12 | -0.14 | 0.03 | 0.14 | **0.63** |
|  | 5 | **-2.55** | -0.33 | -0.13 | -0.14 | 0.03 | 0.15 | **0.64** |
|  | 6 | **-2.23** | -0.13 | -0.12 | **-0.21** | 0.01 | 0.23 | **0.88** |
|  | 7 | **-2.83** | -0.37 | -0.11 | -0.13 | 0.02 | 0.11 | **0.59** |
|  | 8 | **-3.06** | **-0.41** | -0.10 | -0.11 | 0.02 | 0.09 | **0.53** |
|  | 9 | **-2.77** | -0.36 | -0.12 | -0.13 | 0.03 | 0.13 | **0.61** |
|  | 10 | **-4.14** | **-0.65** | -0.05 | -0.03 | 0.02 | -0.04 | 0.26 |
|  | 11 | **-4.03** | **-0.65** | -0.06 | -0.02 | 0.03 | -0.05 | 0.22 |
|  | 12 | **-4.03** | **-0.65** | -0.06 | -0.02 | 0.03 | -0.05 | 0.22 |
|  | 13 | **-4.00** | **-0.65** | -0.06 | -0.02 | 0.03 | -0.04 | 0.23 |
|  | 14 | **-4.30** | **-0.68** | -0.04 | -0.02 | 0.01 | -0.04 | 0.28 |
|  | 15 | **-4.44** | **-0.63** | -0.05 | -0.04 | 0.01 | -0.02 | 0.32 |
|  | 16 | **-4.14** | **-0.64** | -0.05 | -0.03 | 0.02 | -0.04 | 0.26 |
|  | 17 | **-4.35** | **-0.69** | -0.04 | -0.02 | 0.01 | -0.04 | 0.29 |
|  | 18 | **-4.00** | **-0.64** | -0.06 | -0.02 | 0.03 | -0.04 | 0.23 |
|  | 19 | **-3.98** | **-0.64** | -0.06 | -0.03 | 0.03 | -0.04 | 0.23 |
|  | 20 | **-3.98** | **-0.64** | -0.06 | -0.03 | 0.03 | -0.04 | 0.23 |

Note: The bold texts indicate biasedness examined by Welch’s t-test, and the shaded cells biasedness by Yuen’s trimmed mean test at α = 0.05.
