# Supplementary material for: Sample selection bias due to omitting short trees for tree height estimation in forest inventories: A case study on Pinus koraiensis plantations in South Korea
Source: PLoS One. 2025 May 9;20(5):e0321160. doi: 10.1371/journal.pone.0321160 (PMC12063842; doi:10.1371/journal.pone.0321160)
Supplement: S3 Table — (DOCX) [file pone.0321160.s005.docx]

**S3 Table. Variance of HT estimates by model and data type.**

| Model | Full | STF |
| --- | --- | --- |
| 1 | 2.7166 | 1.0665 |
| 2 | 2.4856 | 1.0660 |
| 3 | 2.4699 | 1.0686 |
| 4 | 2.7525 | 1.0589 |
| 5 | 2.7671 | 1.0571 |
| 6 | 2.6340 | 1.1209 |
| 7 | 2.6870 | 1.0668 |
| 8 | 2.5890 | 1.0734 |
| 9 | 2.7378 | 1.0603 |
| 10 | 2.9068 | 1.0664 |
| 11 | 2.7905 | 1.0668 |
| 12 | 2.8157 | 1.0667 |
| 13 | 2.7663 | 1.0669 |
| 14 | 2.8008 | 1.0648 |
| 15 | 2.8215 | 1.0673 |
| 16 | 2.8766 | 1.0667 |
| 17 | 2.7359 | 1.0635 |
| 18 | 2.8417 | 1.0670 |
| 19 | 2.8813 | 1.0671 |
| 20 | 2.8813 | 1.0671 |
