# Supplementary material for: Sample selection bias due to omitting short trees for tree height estimation in forest inventories: A case study on Pinus koraiensis plantations in South Korea
Source: PLoS One. 2025 May 9;20(5):e0321160. doi: 10.1371/journal.pone.0321160 (PMC12063842; doi:10.1371/journal.pone.0321160)
Supplement: S4 Table — (DOCX) [file pone.0321160.s006.docx]

S4 Table. Average changes between HTs estimated from the two datasets by DBH class and model.

| Model | D1 | D2 | D3 | D4 | D5 | D6 | D7 |
| --- | --- | --- | --- | --- | --- | --- | --- |
| 1 | **1.40** | **0.84** | **0.43** | **0.12** | **-0.15** | **-0.36** | **-0.54** |
| 2 | **1.11** | **0.76** | **0.45** | **0.16** | **-0.12** | **-0.36** | **-0.59** |
| 3 | **1.01** | **0.71** | **0.43** | **0.16** | **-0.11** | **-0.34** | **-0.56** |
| 4 | **1.12** | **0.67** | **0.33** | **0.08** | **-0.12** | **-0.26** | **-0.37** |
| 5 | **1.10** | **0.65** | **0.32** | **0.07** | **-0.12** | **-0.25** | **-0.35** |
| 6 | 0.58 | **0.47** | **0.28** | **0.09** | **-0.08** | **-0.21** | **-0.30** |
| 7 | **1.02** | **0.66** | **0.34** | **0.09** | **-0.12** | **-0.27** | **-0.39** |
| 8 | **0.94** | **0.65** | **0.36** | **0.11** | **-0.11** | **-0.28** | **-0.42** |
| 9 | **1.13** | **0.68** | **0.34** | **0.08** | **-0.12** | **-0.27** | **-0.38** |
| 10 | **3.68** | **0.90** | 0.07 | **-0.11** | -0.03 | **0.14** | **0.34** |
| 11 | **3.04** | **1.09** | 0.09 | **-0.20** | **-0.07** | **0.25** | **0.67** |
| 12 | **3.15** | **1.09** | 0.08 | **-0.20** | **-0.06** | **0.27** | **0.69** |
| 13 | **2.90** | **1.08** | **0.12** | **-0.19** | **-0.08** | **0.23** | **0.65** |
| 14 | **3.39** | **1.15** | 0.04 | **-0.23** | -0.04 | **0.32** | **0.72** |
| 15 | **3.63** | **0.99** | 0.07 | **-0.13** | -0.04 | **0.16** | **0.39** |
| 16 | **3.57** | **0.97** | 0.05 | **-0.15** | -0.03 | **0.19** | **0.47** |
| 17 | **3.23** | **1.20** | 0.04 | **-0.26** | -0.03 | **0.36** | **0.78** |
| 18 | **3.31** | **1.00** | 0.06 | **-0.17** | -0.04 | **0.22** | **0.54** |
| 19 | **3.43** | **0.95** | 0.06 | **-0.14** | -0.04 | **0.18** | **0.47** |
| 20 | **3.43** | **0.95** | 0.06 | **-0.14** | -0.04 | **0.18** | **0.47** |

Note: 1) the bold texts indicate the significance of Welch’s t-test at α=0.05, and the shaded cells the significance of Yuen’s trimmed mean test at α=0.05, 2) change = HT estimated from the STF data – HT estimated from the Full data.
